# Effect of Chêneau style braces on vertebral wedging amongst individuals with adolescent idiopathic scoliosis

**DOI:** 10.4102/sajp.v77i2.1617

**Published:** 2021-12-17

**Authors:** Nico Tournavitis, Tuğba Kuru Çolak, Constantinos Voutsas

**Affiliations:** 1 Scoliosis SBPRS, Thessaloniki, Greece; 2 Department of Physiotherapy and Rehabilitation, Faculty of Health Science, Marmara University, Istanbul, Turkey

**Keywords:** adolescent, brace, growth, scoliosis, treatment outcome

## Abstract

**Background:**

It is generally accepted that braces can stop curve progression but little evidence exists regarding structural improvement in the spine using spinal bracing. Our study aimed to investigate the possible structural improvements of vertebral wedging with high correction bracing.

**Objectives:**

The aim of our study was to assess whether spinal brace treatment may influence vertebral wedging in adolescent idiopathic scoliosis (AIS).

**Method:**

We reviewed our database according to the following inclusion criteria: girls with a diagnosis of AIS, Risser 0–2, age 10–14 years with Cobb angles greater than 35°. Our study cohort consisted of 27 patients fulfilling the inclusion criteria with an average brace wearing time of 16.6 h per day and Cobb angles between 36° and 79°. The target value for our study was the apical vertebra wedging, measured twice before brace treatment commenced and twice after the average follow-up period of 20.5 months of treatment.

**Results:**

The average apex wedging noted before brace wearing started was 9.8° (median: 9) and after a period of 20.5 months of brace wearing, it had reduced to an average of 5.8° (median: 4.9), (*p* < 0.001). This would indicate a structural correction of 44%.

**Conclusions:**

Our study supports the hypothesis that spinal high correction braces improve the degree of vertebral wedging in skeletally immature girls with AIS.

**Clinical implications:**

Structural corrections of the apical vertebra seem possible when high correction asymmetric braces are used in the treatment of patients with AIS.

## Introduction

Scoliosis is a three-dimensional (3D) deformity of the spine and trunk (Asher & Burton 2006; Goldberg et al. 2002; Lonstein 1995). Different entities have been described, such as neurogenic, myopathic, congenital types of scoliosis and many others (Chik 2020; Winter 1995). Scoliosis of a known cause may be called symptomatic or syndromic scoliosis (Weiss el al. 2015). Most scoliotic deformities, however, are idiopathic, meaning that there is a deformity in an otherwise healthy child or adolescent (Asher & Burton 2006; Lonstein 1995). Idiopathic scoliosis tends to progress during phases of rapid growth (Asher & Burton 2006; Goldberg et al. 2002; Lonstein 1995; Weiss el al. 2015; Winter 1995). Adolescent idiopathic scoliosis (AIS) (which constitutes 80% – 90% of all types of scoliosis) is the most common form and appears during adolescence between the ages of 10 and 14 years (Asher & Burton 2006; Lonstein 1995; Goldberg et al. 2002). Sometimes, progression in AIS curves during the pubertal growth spurt may happen within a matter of a few weeks (Weiss el al. 2015).

The progression in scoliotic curves is usually explained by the vicious cycle concept with an initial asymmetric loading of the vertebra and disc leading to asymmetric growth. This then may trigger further curve progression (Stokes, Burwell & Dangerfield 2006). However, this concept does not explain why in congenital scoliosis with a calculated risk for progression (Lonstein & Carlson 1984), the curve may stay stable during growth without any treatment, whilst in a patient with AIS, the curve increases (Weiss & Moramarco 2016). Obviously, there are other factors leading to progression in AIS. An explanation may be the concept of functional tethering of the spinal cord as outlined in recent literature (Chu et al. 2006; Deng et al. 2015; Weiss, Moramarco & Borysov 2020).

Nevertheless, when there is a factor triggering curve progression, be it asymmetric loading and/or a functional tether, there may be ways to counteract these forces by correcting the curve to reverse this vicious cycle (Landauer, Wimmer & Behensky 2003; Weiss & Kleban 2015; Weiss el al. 2015, 2017). In the last decade, this has been undertaken by making use of high correction asymmetric Chêneau style braces (Landauer et al. 2003; Weiss & Kleban 2015; Weiss el al. 2015, 2017). Although there are authors reporting on an improvement of back shape and trunk (Weiss & Moramarco 2013; Weiss 2014), as well as authors showing that the angle of curvature can be improved with the help of Chêneau style braces (Landauer et al. 2003; Weiss 2014; Weiss & Kleban 2015; Weiss & Moramarco 2013; Weiss el al. 2015, 2017), a systematic investigation of possible structural improvements of vertebral wedging has not yet been undertaken.

Little (2016) has shown that there is an overall pattern of increased disc wedging near the apex of the curvature and reduced joint compliance in this region. Furthermore, Sun (2018) found the same segmental characteristics of main thoracic curves in patients with severe AIS. In the latter study, the apical and adjacent vertebrae accounted for 67.44% ± 8.05% of the vertebral wedging of the whole curve. During curve progression, if structural changes arise (leading to a wedging of the vertebrae and disc spaces at the apical and adjacent area of the curve), it should be possible to reverse these structural changes in skeletally immature patients with good in-brace correction. The purpose of our study was to investigate if Chêneau style brace treatment may impact vertebral wedging in immature patients with single thoracic curve patterns and curvatures exceeding 35°.

## Method

Our study was conducted in compliance with the Declaration of Helsinki. A parent of each child was informed about the study and written consent was obtained from the participants.

This was a cross-sectional retrospective study, and a quota sampling technique was used to select patients. Patients who were included had participated in Scoliosis Best Practice Rehabilitation Services Thessaloniki, Athens, and Nicosia, between June 2014 and September 2017. Patients were routinely monitored whilst wearing the brace.

Patients enrolled in our study continued to wear a brace after our study was completed. The results of the X-ray examinations of the first and last follow-up are presented here and the X-ray examinations were conducted without the brace being worn.

For each new brace, X-ray check-ups were carried out with the brace being worn for 6 weeks after the adjustment. X-ray examinations were only initiated if a new brace was necessary. All patients included in our study had at least two X-rays without a brace (performed to design new braces). Some patients received three braces maximum.

**FIGURE 1:**
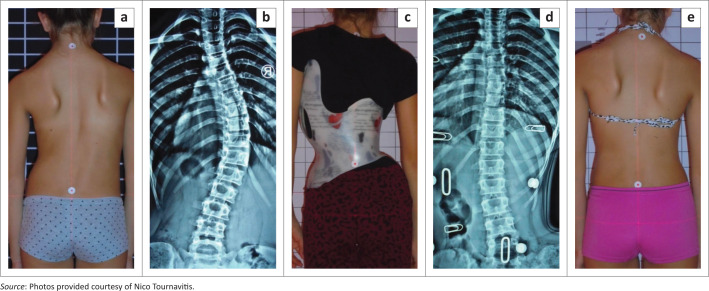
Clinical and radiological intermediate result after 6 weeks. From left to right: (a) initial clinical appearance of a single thoracic scoliosis with (b) 44° of Cobb angle. (c) Patient in brace mirroring of initial deformity is clearly visible. (d) In-brace X-ray with –17° of Cobb angle with a (e) clinical over-correction of the pelvic and the trunk.

We reviewed all radiographs of patients with AIS and a single thoracic curve pattern with a Cobb angle (Cobb 1948) greater than 35° from our clinical database. The demographic characteristics of patients are presented in Table 1. All patients had a standardised computer-aided designed Chêneau style brace (Gensingen brace/GBW^®^, see also Figure 1). Further inclusion criteria were girls having a Risser sign of 0–2 aged 10–14 years with a single thoracic curve pattern. These criteria were selected as single thoracic curve patterns are easier to correct than combined curve patterns (Weiss et al. 2020). Therefore, we estimated that it would be easier to show structural changes in this specific curve pattern. The exclusion criteria were as follows: non-idiopathic scoliosis, neuro-muscular or rheumatic diseases, a previous spinal operation or having received other treatment for scoliosis.

**TABLE 1:** Demographic and clinical characteristics of patients (*n* = 27).

Variables	Mean ± SD	Median	min–max (*n* = 27 female)
Age (years)	11.8 ± 1.1	11.8	10.1–13.8
Risser sign	0.5 ± 0.7	0	0–2
Number of vertebrae in the curve	6.8 ± 1.1	7	5–9
Apex of the curve	*T 8.3 ± 0.8	T 8	T 7–10

*Source*: Table provided by the second author, Tuğba Kuru Çolak.

* T, Thoracic.

In this curve pattern, there is a long structural C-curve. Long lever arms enable good corrections and may have a beneficial impact on the apical area of the curve. As structural changes are only possible during growth, we only included adolescent patients with the first signs of maturation who were in the pubertal growth spurt.

### The brace used

The brace used for these patients was a Gensingen brace (GBW), which is a Chêneau derivative. Chêneau derivates are asymmetrical trunk orthoses adapted to the corresponding curvature pattern, which today are still often made from a plaster cast. Computer-aided design (CAD)/computer-aided manufacturing (CAM) brace libraries are monitored by experts and continuously being improved, allowing the brace standard to be improved overall (Weiss et al. 2021). Internationally, the GBW use is widespread. The GBW was introduced in 2010 (Weiss 2010) and since 2015 has been used with continuous development and evaluation by international specialists (Weiss et al. 2021) at the first author’s centre (Figure 1).

### Target value: Apical vertebra wedging measured according to Cobb

The Cobb method was used to measure the degree of scoliosis: vertical lines were drawn at the upper and lower end vertebra endplate lines on the whole spine anteroposterior (AP) X-ray film and included the angle of the two vertical lines.

The apical vertebra is defined as the most deviated vertebra from the vertical axis that passes through the patient’s sacrum on the AP view. The apical vertebra of a scoliotic curve is the most wedged vertebra, and the amount of wedging is bigger in curvatures exceeding 30° (Modi et al. 2008). The apical vertebra wedging (AVW) was determined before starting brace treatment and after the follow-up period.

The AVW angle was measured with the angle formed by two lines connecting the upper and lower endplate of the vertebrae and was made using the Surgimap® software (Modi et al. 2008). All measurements were taken twice by the first author to ensure good intra-rater reliability.

Because the AP measurement of the Cobb angle on an X-ray is a two-dimensional measurement only and because the Cobb angle would be significantly bigger in the ‘Plan d‘election’ (when the scoliotic curve is rotated into the frontal plane; Deacon, Archer & Dickson 1987; Dickson 2011; Stagnara 1965), a second value might be necessary to exclude any artefacts of measuring by changes in rotation. According to the authors (Deacon et al. 1987; Dickson 2011; Stagnara 1965), the Cobb angle is the biggest when the trunk is turned into the plane of rotation (Plan d‘election). This would mean that when rotation is just decreased by simply turning the trunk (Spine) in front of the X-ray detector without changing the Cobb angle, the measurement of the projected Cobb angle would be bigger than in the frontal AP view on the patient’s trunk (Dickson 2011). Therefore, a correction of the Cobb angle together with a correction of the rotation can be estimated to be a true reduction of the Cobb angle, not compromised by a rotation artefact.

We only considered readable X-rays and measurements taken at the upper and lower endplate of well-defined vertebrae. Therefore, most of the variables contributing to the usual technical error of the Cobb angle measurements do not apply for the measurements taken in our study.

For this reason, we introduced the angle of trunk rotation (ATR) measurements according to Bunnell (2005) with the help of the Scoliometer™ as a secondary target value (Weiss et al. 2021) to reduce projection artefacts because of an increase or decrease in trunk rotation. The ATR value was obtained in a forward bending position, and the maximum angle as measured was analysed. Given that this was a retrospective study, these measurements were recorded as part of our patients’ management programme and then analysed for our study.

### Statistical analysis

Data analysis was performed using Statistical Package for the Social Sciences (SPSS) version 16. The Shapiro-Wilk test was used to test the normality of each variable; *p*-values less than 0.05 were considered to be statistically significant for a two-tailed test. Baseline and last follow-up measurements were compared using the Wilcoxon signed-rank test. Intra-rater reliability was established by Spearman’s correlation coefficient.

Structural correction was computed simply. The last measured AVW value was subtracted from the first AVW measurement. The percentage of the obtained (result of subtraction) value was calculated.

## Results

**FIGURE 2:**
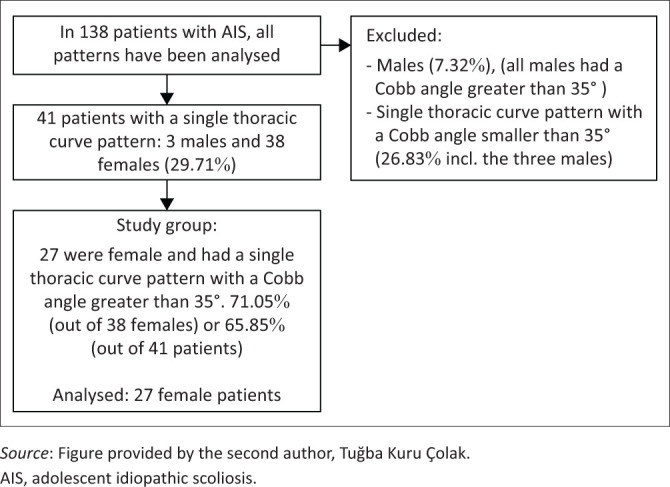
Flow chart of participants.

A total of 27 female patients from our database fulfilled the inclusion criteria (Figure 2). Patients’ mean age was 11.8 years and mean Risser sign value was 0.5 (Table 1). Their mean Cobb angle was 43.9º (range 36º–79°). The average number of vertebrae in the curve was 6.8, and the average apex of the curve was T 8.3. Cobb angle and ATR both improved significantly (Table 2).

**TABLE 2:** Mean change in Cobb angle, apical vertebra wedging and angle of trunk rotation (*n* = 27).

Variables	Before treatment			After monitoring period			Mean differences			*p*	*Z*
	Mean ± SD	Median	min–max	Mean ± SD	Median	min–max	Mean ± SD	Median	min–max		
Cobb angle (°)	43.9 ± 12.7	38	36–79	34.7 ± 17.2	34	9–74	−9.1 ± 9.5	−7	−38–8	< 0.001	−3.871
Apical vertebra wedging (°)	9.8 ± 4.6	9	3–19	5.8 ± 4.2	4.9	0.4–14.6	−3.9 ± 2.6	−4.2	−10.1–1.0	< 0.001	−2.480
ATR angle (°)	12.8 ± 4.9	11	7–26	10.6 ± 5.7	10	1–25	−2.1 ± 4.1	−2	−13–6	0.013	−4.382

*Source*: Table provided by the second author, Tuğba Kuru Çolak.

ATR, angle of trunk rotation.

**FIGURE 3:**
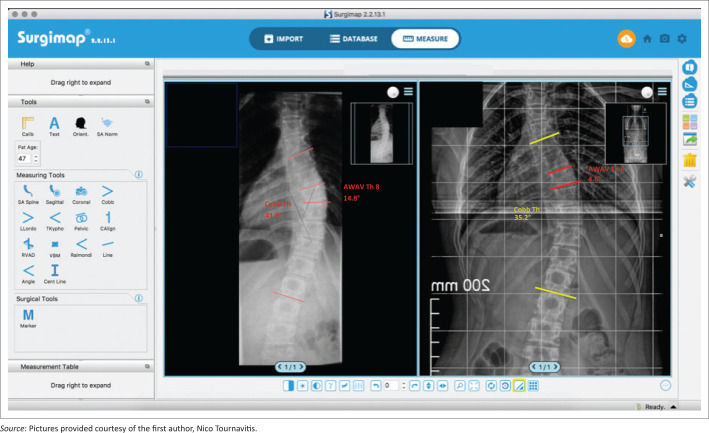
Reduction of the apical vertebra angle because of the structural correction of the apical and adjacent vertebra after pattern-specific computer-aided design Chêneau style bracing. A reduction has been found in this case. The apical vertebra decreased from 14.8° to 4.6° after 11 months of treatment (Risser 0). The most wedged vertebrae were above the apex in this case.

Apical vertebra wedging before bracing was 9.8° and after the monitoring period it was 5.8° (*p* < 0.001). The monitoring period was an average of 20.5 months (range 11–30 months). These results are statistically significant, demonstrating a structural correction within the apical vertebra of 44% (*p* < 0.001) (Table 2 and Figure 3 – Figure 5).

**FIGURE 4:**
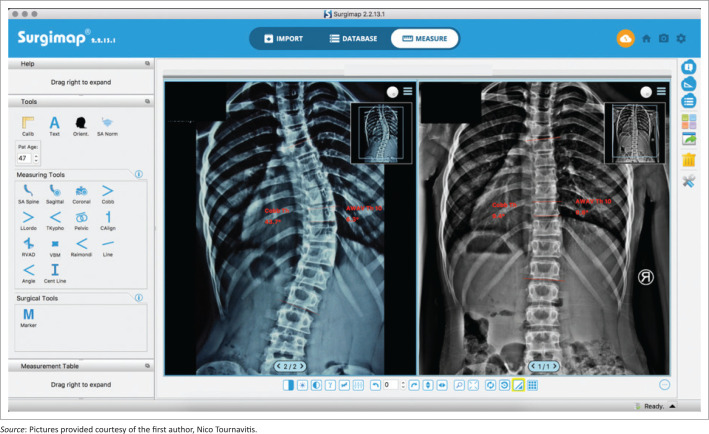
This case shows that with highly corrective asymmetric Chêneau style braces (Gensingen Brace®) structural corrections are possible and favourable final outcomes for the patient can be achieved. Almost complete restoration with the wedged vertebra changing from 6.3° to 0.6° after 10.5 months of treatment (Risser 0).

**FIGURE 5:**
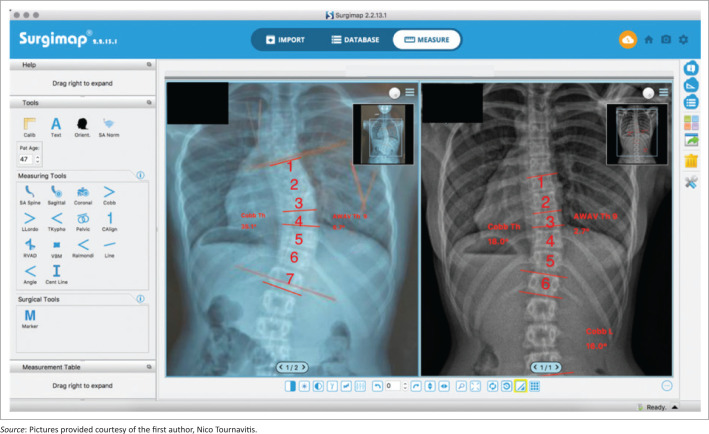
Reduced number of vertebrae affected after 14 months of treatment, from seven to six vertebrae (Risser 0) within the curve.

The intra-rater correlation of the measurement of AVW was *r* = 0.99; *p* < 0.05 with the technical error ranging from 0º to 1.3°. Therefore, the differences as found at average clearly exceed the maximum technical error. The brace wearing time as reported by the patients and their parents was 16.9 h/day (ranged: 8 to 22 h).

## Discussion

Our study results suggest that wearing a reliable high correction Chêneau style brace (Gensingen Brace GBW^®^) may lead to the reduction of the structural deformity of the apical vertebrae. This may explain the stable corrections after brace weaning in a high percentage of patients treated with high correction braces (Aulisa et al. 2017; Weiss et al. 2021).

The coincidence of a correction of the Cobb angle and of the rotation (ATR) after the follow-up period again suggests that the improvements of AVW are real structural improvements of the bony shape of the vertebral body and not because of a positional artefact. As the Cobb angle would be significantly bigger in the Plan d‘election (when the scoliotic curve is rotated into the frontal plane where the rotation of the apical vertebra would be 0°; Deacon et al. 1987; Dickson 2011; Stagnara 1965), the Cobb angle measurement would be expected to be smaller when the scoliotic curve is measured in the standard AP X-ray position whilst the rotation of the apical vertebra increases. Therefore, when there is simultaneously a decrease in rotation and a decrease in Cobb angle, we may conclude that a real structural improvement has been achieved, not compromised by a rotational artefact.

The vertebral wedging is seen as the most important factor in progression of scoliosis by some researchers (Braun et al. 2003; Perdriolle et al. 1993). The latter authors (Perdriolle et al. 1993) have underlined that vertebral wedging is not only the most essential deformation process, but also has a direct correlation with the curve magnitude. According to the Hueter-Volkmann law (Mehlman, Araghi & Roy 1997), compressive forces inhibit the epiphyseal growth, and distraction forces stimulate the epiphyseal growth. Based on this law, Roaf (1960) has suggested a ‘vicious cycle’ concerning kyphosis progression. According to this concept, a minimal wedging of the vertebrae would produce asymmetric compressive force on the vertebral end plates, which would further increase the wedging as per the Hueter-Volkmann’s law and thus produce further abnormal forces. For this reason, it is important to control vertebral wedging within the conservative management of spinal deformities.

We analysed the radiographs of patients with Cobb angles exceeding 35°, and the mean AWV value was 9.8° before treatment. Modi et al. (2008) evaluated the radiographs of patients with AIS with a Cobb angle of 10° – 60° (mean: 27.1) and an age range of 11–20 years, and reported 4.1° of vertebral body wedging in the thoracic spines of patients with Cobb angles above 30°. However, the authors did not provide a study in a pre-/post-design outlining an effect of any treatment. The difference in AWV between our study and that of Modi et al. (2008) could be explained by the difference in the mean Cobb angles. To the best of our knowledge, there is no study investigating the effect of brace treatment on vertebral wedging.

Structural corrections of the apical vertebra seem possible when high correction asymmetric braces are used in the treatment of patients with AIS. Structural corrections of the apical and adjacent vertebrae have a favourable influence on the outcome for the patient. It has been found that in a reasonable percentage of patients treated with a high correction brace, final improvements can be achieved (Landauer et al. 2003; Weiss 2014; Weiss & Kleban 2015; Weiss & Moramarco 2013; Weiss, Seibel & Kleban 2014; Weiss et al. 2017, 2019; ). As AIS does not lead to severe health problems besides the deviation (Weinstein, Zavala & Ponseti 1981; Weinstein et al. 2003; Weiss et al. 2016), the focus in future studies should shift from aiming at improvements of Cobb angle to improvement of the cosmetic outcomes and how different braces may influence these in the long term.

We used a standardised computer-aided designed Chêneau style brace (GBW^®^). In a certain percentage of patients using the GBW, improvements of their spinal deformity have been noted in addition to improvement of the Cobb angle (Weiss & Moramarco 2013; Weiss 2014; Weiss et al. 2014, 2019). Within this investigation, we found structural changes explaining the improvements obtained in Cobb and ATR angles.

Our study focused on one single curve pattern selectively to see whether structural changes are possible at all when applying asymmetric braces. In a study by Borysov et al. (2013), the in-brace correction in double curve patterns was less than that in single curve patterns. Therefore, in future studies, the effect on bracing in combined curves should be investigated.

For symmetric braces (Weinstein et al. 2013), structural remodelling effects have not been found. Therefore, we would suggest applying standardised CAD Chêneau derivates wherever possible (Rigo 1999, Rigo 2003; Rigo & Weiss 2003; Weiss 2014; Weiss & Moramarco 2013; Weiss et al. 2017).

The technical error (variability of the measurements) of the Cobb angle measurement lies between 3° (Wang et al. 2018) and 6° (Loder et al. 2004). Therefore, measurement differences of ± 5° are defined as unchanged, whilst for the definition of improvement or progression, a difference of 6° or more Cobb angle is defined. This would be appropriate for measuring whole curvatures involving six or more vertebrae. Most of the technical error may be because of the variability of segment selection, variability in the selection of upper or lower endplate of the vertebra and lack of readability of the X-ray (quality of the picture).

Studies evaluating the effect of brace treatment on AVW in patients with AIS are very limited. Presenting the results of AVW assessment in patients with a specific curve pattern and continued growth and with risk of progression is the strength of our study.

The lack of blind *assessors* collecting outcome data and comparison of the effect of different brace types on AVW is a limitation of our study. Secondly, the AVW as measured according to Cobb on an AP X-ray is a measurement in one plane only and does not describe the true 3D character of a scoliotic deformity. A complete 3D analysis would only be possible with the help of the 3D reconstruction of a computer tomography scan. However, the radiation exposure resulting from this examination does not seem appropriate to the question of our examination.

## Conclusion

Our study supports the hypothesis that spinal high correction braces may improve the degree of vertebral wedging in skeletally immature girls with AIS.
